# Cultivated and wild pearl millet display contrasting patterns of abundance and co-occurrence in their root mycobiome

**DOI:** 10.1038/s41598-021-04097-8

**Published:** 2022-01-07

**Authors:** Marie-Thérèse Mofini, Abdala G. Diedhiou, Marie Simonin, Donald Tchouomo Dondjou, Sarah Pignoly, Cheikh Ndiaye, Doohong Min, Yves Vigouroux, Laurent Laplaze, Aboubacry Kane

**Affiliations:** 1 Département de Biologie Végétale, Faculté des Sciences et Techniques, Université Cheikh Anta Diop (UCAD), BP 5005, Dakar Fann, Senegal; 2 Laboratoire Mixte International Adaptation des Plantes et Microorganismes associés aux Stress Environnementaux (LAPSE), Centre de recherche de Bel-Air, Dakar, Sénégal; 3 Laboratoire Commun de Microbiologie (LCM), Centre de Recherche de Bel-Air, Dakar, Sénégal; 4 Centre d’Excellence Africain en Agriculture pour la Sécurité Alimentaire et Nutritionnelle (CEA-AGRISAN), UCAD, Dakar, Sénégal; 5 Centre d’Etude Régional pour l’Amélioration de l’Adaptation à la Sécheresse (CERAAS), Institut Sénégalais de Recherches Agricoles (ISRA), Route de Khombole, Thiès, Sénégal; 6 IPME, IRD, Cirad, Université de Montpellier, Montpellier, France; 7 DIADE, Université de Montpellier, IRD, Cirad, 911 Avenue Agropolis, 34394 Montpellier cedex 5, France; 8 Department of Agronomy, Kansas State University, Manhattan, KS USA; 9 Present Address: Université d’Angers, Institut Agro, INRAE, IRHS, SFR 4207 QuaSaV, 49000 Angers, France

**Keywords:** Microbiome, Soil microbiology

## Abstract

Fungal communities associated with roots play a key role in nutrient uptake and in mitigating the abiotic and biotic stress of their host. In this study, we characterized the roots mycobiome of wild and cultivated pearl millet [*Pennisetum glaucum* (L.) R. Br., synonym: *Cenchrus americanus* (L.) Morrone] in three agro-ecological areas of Senegal following a rainfall gradient. We hypothesized that wild pearl millet could serve as a reservoir of endophytes for cultivated pearl millet. We therefore analyzed the soil factors influencing fungal community structure and whether cultivated and wild millet shared the same fungal communities. The fungal communities associated with pearl millet were significantly structured according to sites and plant type (wild vs cultivated). Besides, soil pH and phosphorus were the main factors influencing the fungal community structure. We observed a higher fungal diversity in cultivated compared to wild pearl millet. Interestingly, we detected higher relative abundance of putative pathotrophs, especially plant pathogen, in cultivated than in wild millet in semi-arid and semi-humid zones, and higher relative abundance of saprotrophs in wild millet in arid and semi-humid zones. A network analysis based on taxa co-occurrence patterns in the core mycobiome revealed that cultivated millet and wild relatives had dissimilar groups of hub taxa. The identification of the core mycobiome and hub taxa of cultivated and wild pearl millet could be an important step in developing microbiome engineering approaches for more sustainable management practices in pearl millet agroecosystems.

## Introduction

Climate change poses new threats to food security as the human population continues to grow^1^, and the price of fertilizers continues to rise. A paradigm shift is needed to address future challenges faced by agriculture. One such potential change would be to harness the potential of plant microbiomes and especially root-associated fungi to improve plant nutrition and health. Root-associated fungi are a diverse group of microorganisms that develop on the surface or in the tissues of the host. Through the interaction with their host, some of these fungi play beneficial roles that contribute to the health of their host. For instance, they can produce bioactive secondary metabolites that can protect their host against pathogen and insect attacks and enhance abiotic stress tolerance^2–4^. They also promote the water and minerals absorption by their host^5^. On the other hand, some of these fungi, especially pathogens, can strongly reduce their host productivity under certain conditions^6,7^.

In cereals, root-associated fungi belong to taxonomically diverse groups including mycorrhizal fungi, mainly arbuscular mycorrhizal fungi (AMF), and non-mycorrhizal fungi^8–11^. The latter group is dominated by endophytic fungi which have different lifestyles (mutualistic, latent pathogen and latent saprophyte) depending on host genotype and physiology (senescence, flowering, fruition, vegetative period and age^12–14^). According to Nguyen et al.^15^ these root-associated fungi can be classified into three main groups based on their trophic modes: (1) symbiotroph, receiving nutrients by exchanging resources with host cells; (2) pathotroph, receiving nutrients by harming host cells; and (3) saprotroph, receiving nutrients by breaking down dead host cells. It is important to note, however, that many fungal genera contain more than one trophic strategy^16^. These fungal genera have been considered as multi-trophic mode groups^17^. Through their different trophic modes, the root-associated fungi can interact directly or indirectly with each other positively or negatively, and thereby influence the fitness of their host plant^18–20^. On the other hand, it has been established that host genotype and environment factors shape microbiome assembly in natural environments^20–22^.

Pearl millet [*Pennisetum glaucum* (L.) R. Br., synonym: *Cenchrus americanus* (L.) Morrone] is a major staple food and source of fodder and fuel in the arid and semi-arid regions of sub-Saharan Africa and India. The vegetative, reproductive and physiological characteristics of pearl millet make it a crop well suited for growth under difficult conditions, including low soil fertility, high pH, low soil moisture, high temperature, high salinity and limited rainfall where other cereals such as maize, rice, sorghum or durum wheat would fail^23^. Pearl millet grain content is highly nutritious, with 8–19% protein, low starch levels, high fiber content (1.2 g/100 g^24^), and higher micronutrient concentrations (iron and zinc) than rice, wheat, maize and sorghum^25^. It was domesticated in the central Sahel (Mali-Niger) about 4900 years ago as corroborated by archaeological and genomic studies^26^.

In Senegal, cultivated pearl millet still coexists with wild millet around or even inside some farmers’ plots. Gene flows with wild relatives in the western and eastern Sahel are important and have contributed to increase the diversity of cultivated pearl millet in Africa^26^. However, the comparison of the microbiomes of cultivated and wild plants has not yet been performed. We hypothesized that wild pearl millet could act as a potential microbial reservoir for cultivated plants. In addition, understanding the complex fungal community assemblage of cultivated and wild millet could help to develop more sustainable management practices in pearl millet agroecosystems.

Here, we employed DNA metabarcoding techniques to characterize fungal communities associated with cultivated and wild pearl millet roots in three agro-ecological zones of Senegal following a rainfall gradient. We examined the influence of soil parameters on the structure of fungal communities and identified the pearl millet core mycobiome common to wild and cultivated plants across the three zones. Identification of this core mycobiome might provide information on fungi potentially involved in maintaining community stability and which may play an important role in plant health and productivity. Network analysis was then performed to investigate co-occurrence patterns and identify potential hub taxa in the core mycobiome of cultivated and wild plants. Once again, this approach should provide potential target hub groups for further studies and for engineering approaches.

## Results

### Experimental sites have contrasted soil properties

In order to analyze the fungal communities associated with millet roots and the factors driving their structure, we first characterized the soil properties of the experimental plots from three agro-ecological zones [Darou-Mousty (arid zone), Dya (semi-arid zone) and Nioro (semi-humid zone)] in Senegal. Statistically significant differences in soil properties [pH, total nitrogen (N), total phosphorus (P), carbon to nitrogen ratio (C:N) and ammonium (NH_4_^+^)] were found between the three zones (Supplementary Table S1). When the properties of soils collected under cultivated plants were compared to those of soils collected under wild plants, we observed significant differences for the carbon:nitrogen (C:N) ratio which was higher in soil collected under wild plants in Dya (*p* = 0.036), and Nioro (*p* = 0.022), total P which was higher in soil collected under cultivated plants in Nioro (*p* = 0.002), and pH H_2_O which was lower in soil collected under cultivated plants in Nioro (*p* = 0.005).

### Wild and cultivated pearl millet have different taxonomic diversity and composition of root-associated fungal communities

**Figure 1:**
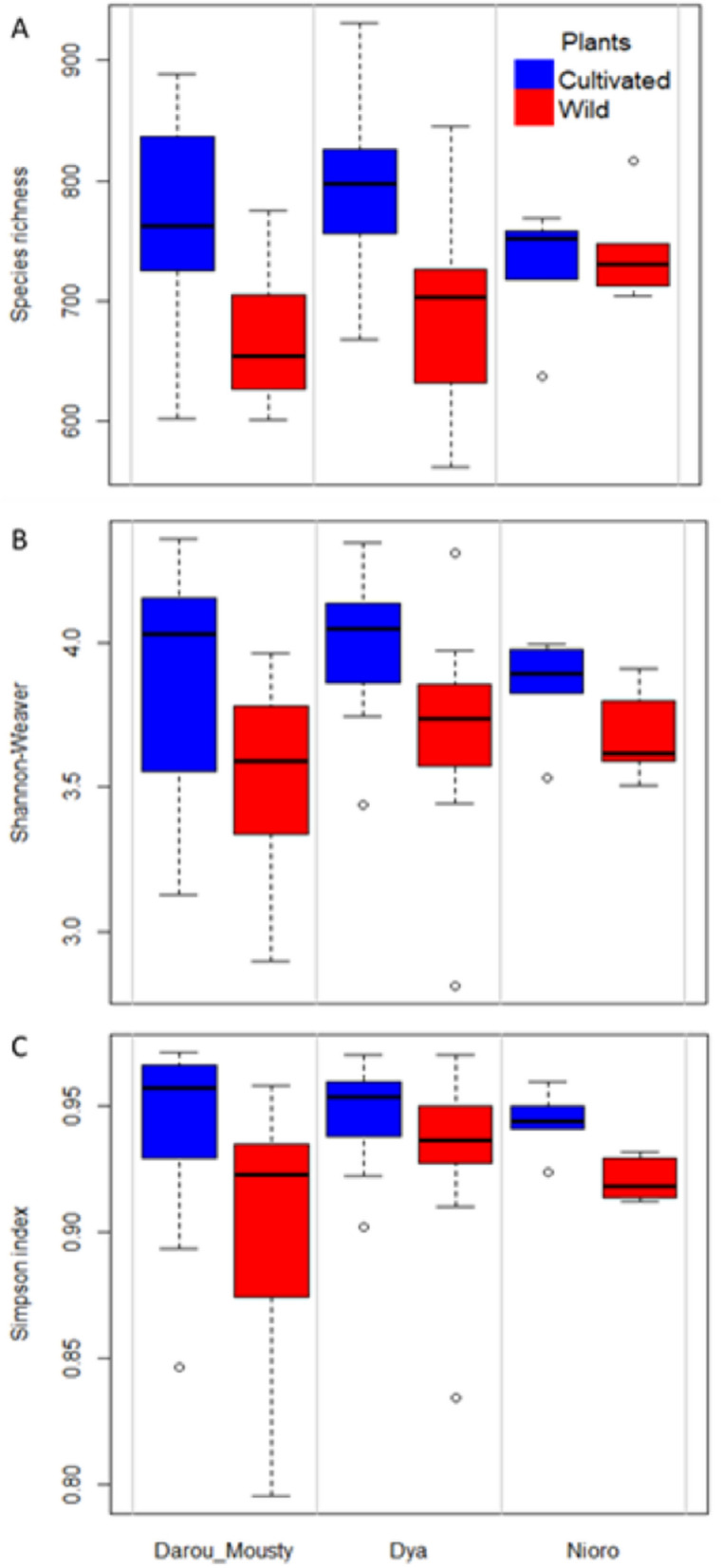
Species richness (**A**) and Shannon diversity index (**B**) of the fungal communities associated with cultivated and wild pearl millet in the three studied sites (Darou-Mousty, Dya and Nioro). In the linear mixed effects (LME) model used to test the effect of plant type and site, plot was included as a random factor (see Table 1).

We used metabarcoding targeting the internal transcribed spacer 1 (ITS1) region to characterize the fungal communities associated with cultivated and wild millet roots. After sequencing, the normalized dataset accounted for 5524 fungal operational taxonomic units (OTUs) for wild and cultivated pearl millet. The site had no effect on fungal species richness, Shannon index and Simpson diversity index (Fig. 1A–C, Supplementary Table S2). However, the plant type (cultivated vs wild) had a significant effect on all the indices studied (species richness, *p* = 0.000; Shannon, *p* = 0.000; Simpson, *p* = 0.009). In addition, the variance explained by the random factor (plot, R^2^c − R^2^m) was low compared to those explained by the fixed factors (plant type and site, R^2^m) for the different α-diversity indices (Supplementary Table S2). Hence, the various α-diversity indices revealed that cultivated pearl millet display a higher fungal diversity than wild pearl millet.

**Table 1:** Results from ANOVA of the linear mixed effects (LME) model testing the effect of plant type, site and their interaction on species richness, Shannon and Simpson diversity indexes of the fungal communities associated with cultivated and wild Pearl millet.

Factors	numDF	denDF	F	*p*	R^2^m	R^2^c
**Richness**						
Intercept	1	51	6450.426	< 0.0001	0.310	0.310
Plants	1	51	19.153	0.000		
Sites	2	3	0.936	0.483		
Plants:Sites	2	51	2.736	0.074		
**Shannon**						
Intercept	1	51	5988.246	< 0.0001	0.216	0.258
Plants	1	51	14.182	0.000		
Sites	2	3	0.755	0.542		
Plants:Sites	2	51	0.321	0.727		
**Simpson**						
Intercept	1	51	32,490.240	< 0.0001	0.162	0.209
Plants	1	51	7.470	0.009		
Sites	2	3	1.100	0.439		
Plants:Sites	2	51	0.530	0.591		

R^2^m (marginal r squared) represents the variance explained by the fixed factors, and R^2^c (conditional r squared) represents the variance explained by the both fixed and random factors.

**Figure 2:**
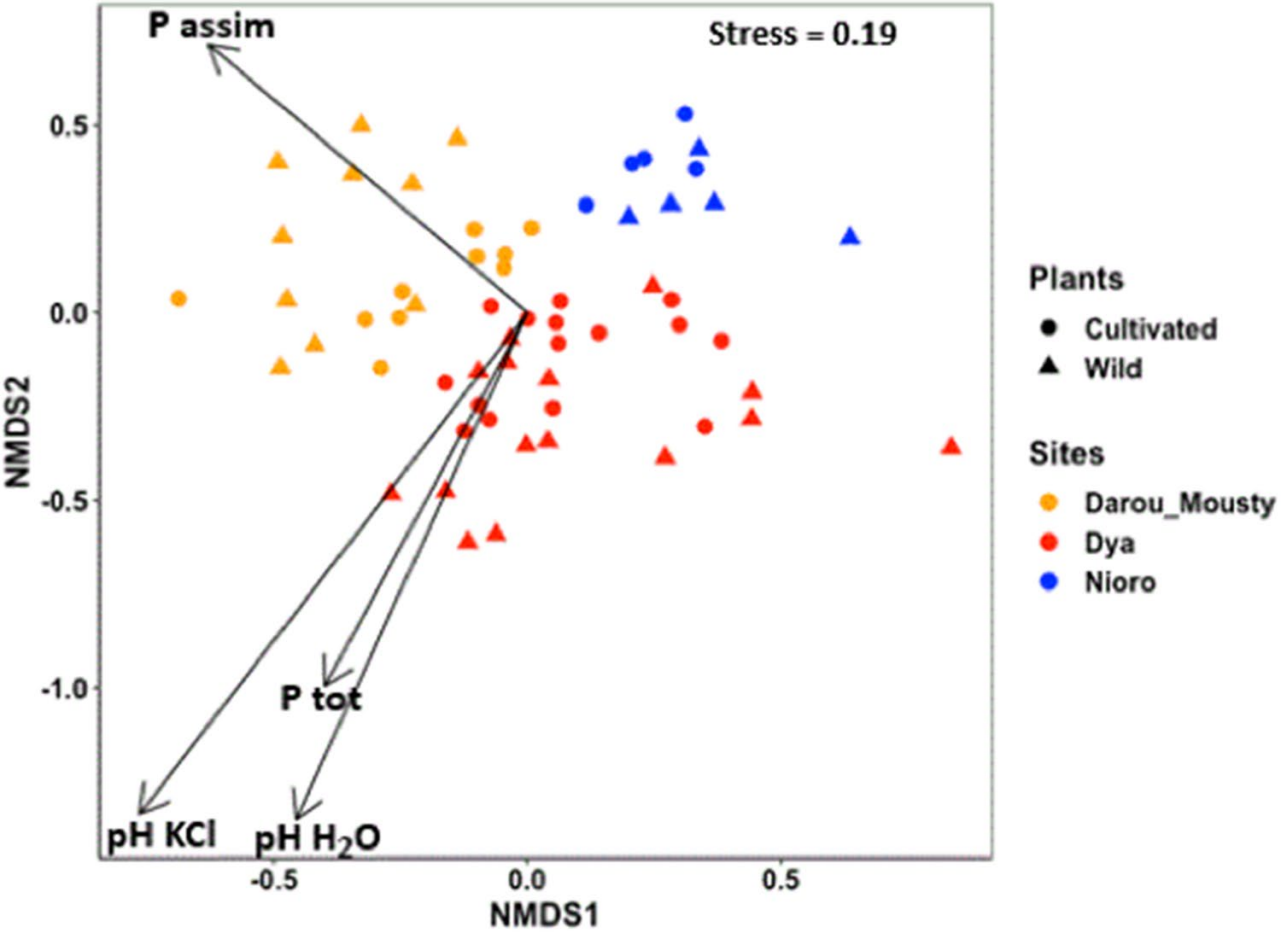
Non-Metric Multidimensional Scale (NMDS) plots depicting the similarity of fungal communities according to sites and plant types, and the relative importance of soil properties (arrows) explaining the variation in fungal communities according to sites. Only significant factors (*p* < 0.05) correlated with fungal community dissimilarity are presented. Each point represents a single sample.

The non-metric multidimensional scaling (NMDS) allowed us to visualize the distribution of our samples in the two-dimensional space (stress value of 0.19). We observed that site and plant type and their interaction were the major factors structuring fungal communities in the data set, while the effects of plant type and site-plant type interaction were minor (Table 2, Supplementary Table S3, Fig. 2). The root fungal communities were distributed along the first two dimensions of the NMDS based on the site where their plant host inhabits, with a separation between the samples from Darou-Mousty and those from Nioro across the axis 1, and the samples from Dya and those from Nioro across the axis 2 (Fig. 2). The analysis indicated that variation in fungal community structure was mainly explained by soil pH and phosphorus (Fig. 2; Supplementary Table S4).

**Table 2:** Summary of non-parametric permutational multivariate analysis of variance (PERMANOVA) based on Bray–Curtis distance to test the effects of site and plant type on the structure of fungal communities associated to pearl millet roots.

Factors	*df*	SS	MS	F.Model	R^2^	*p* value
Sites	2	3.1885	1.59425	7.9021	0.21097	0.001
Plants	1	0.4361	0.43606	2.1614	0.02885	0.001
Sites × plants	2	0.5947	0.29737	1.4739	0.03935	0.005
Residuals	54	10.8945	0.20175	0.72083		
Total	59	15.1138	1.00000			

*df*  degrees of freedom, *SS* sum of squares, *MS* mean sum of squares, *F* *model* *F* statistics, *R*^2^ partial R-squared, based on 999 permutations.

**Figure 3:**
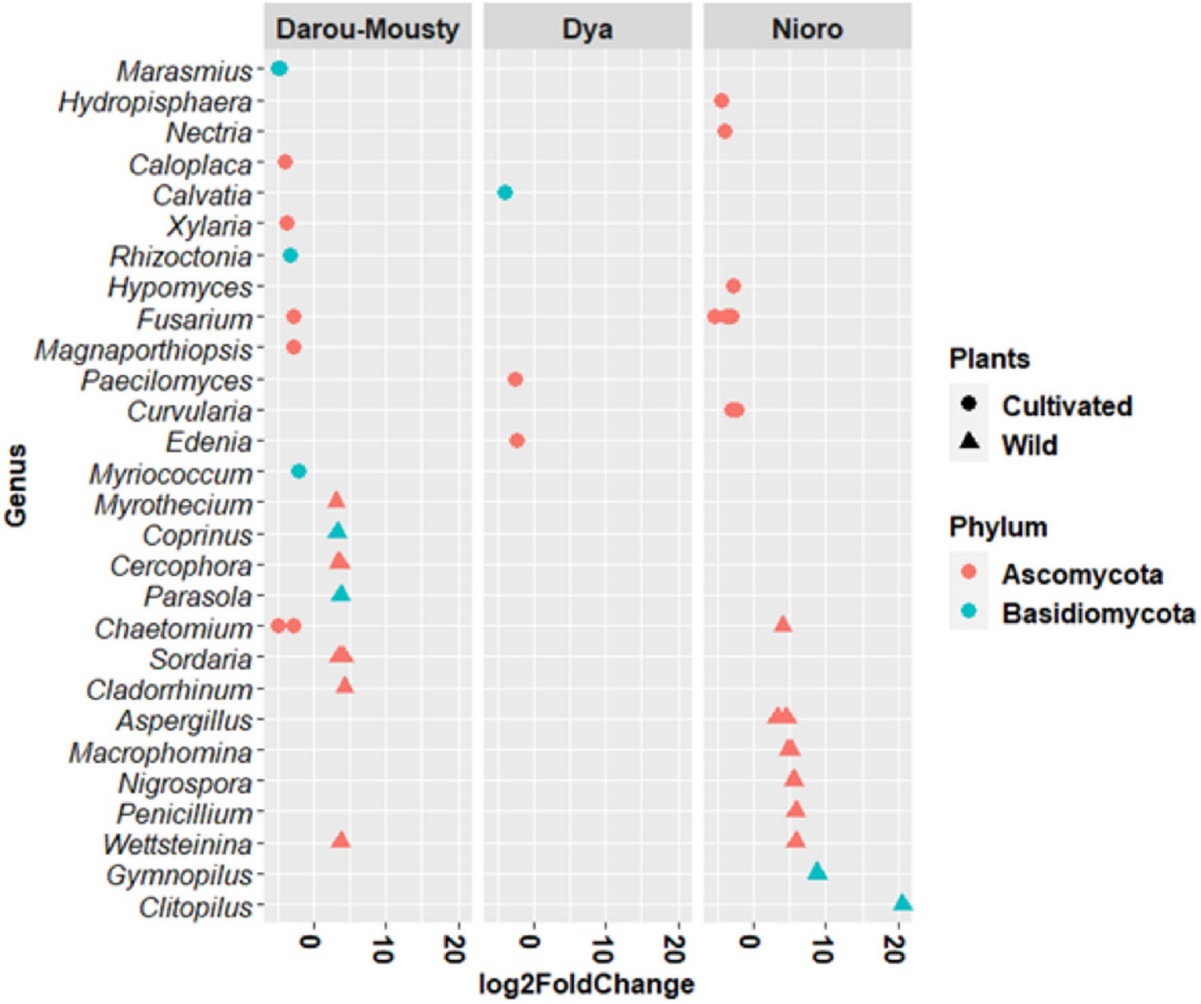
Differentially abundant OTUs detected in cultivated and wild Pearl millet by pairwise comparison (DeSeq2 analysis of 5524 OTUs, with *p* value adjusted to the 1% threshold). Each point represents an individual OTU, which was assigned to genus (y-axis, 63 OTUs) and phylum level (colours). In the x-axis, positive values of log2 fold change indicate higher abundance of OTUs in wild plants and negative values indicate higher abundance of OTUs in cultivated plants.

In Darou-Mousty, 19 OTUs were differentially abundant based on plant type, of which 9 OTUs were significantly enriched in wild plants and 10 enriched in cultivated plants (Fig. 3). In Nioro, 12 OTUs were enriched in wild plants and 9 were enriched in cultivated plants whereas only 3 OTUs were significantly enriched in cultivated plants in Dya (Fig. 3). On the other hand, only the genera *Fusarium* and *Chaetomium* had differentially abundant OTUs in more than one site (Darou-Mousty and Nioro). The mean abundances and log twofold change values of differentially abundant OTUs in each site and plant type are given in Supplementary Table S5.

### Wild and cultivated pearl millet show differences in hosted fungal functional groups

**Figure 4:**
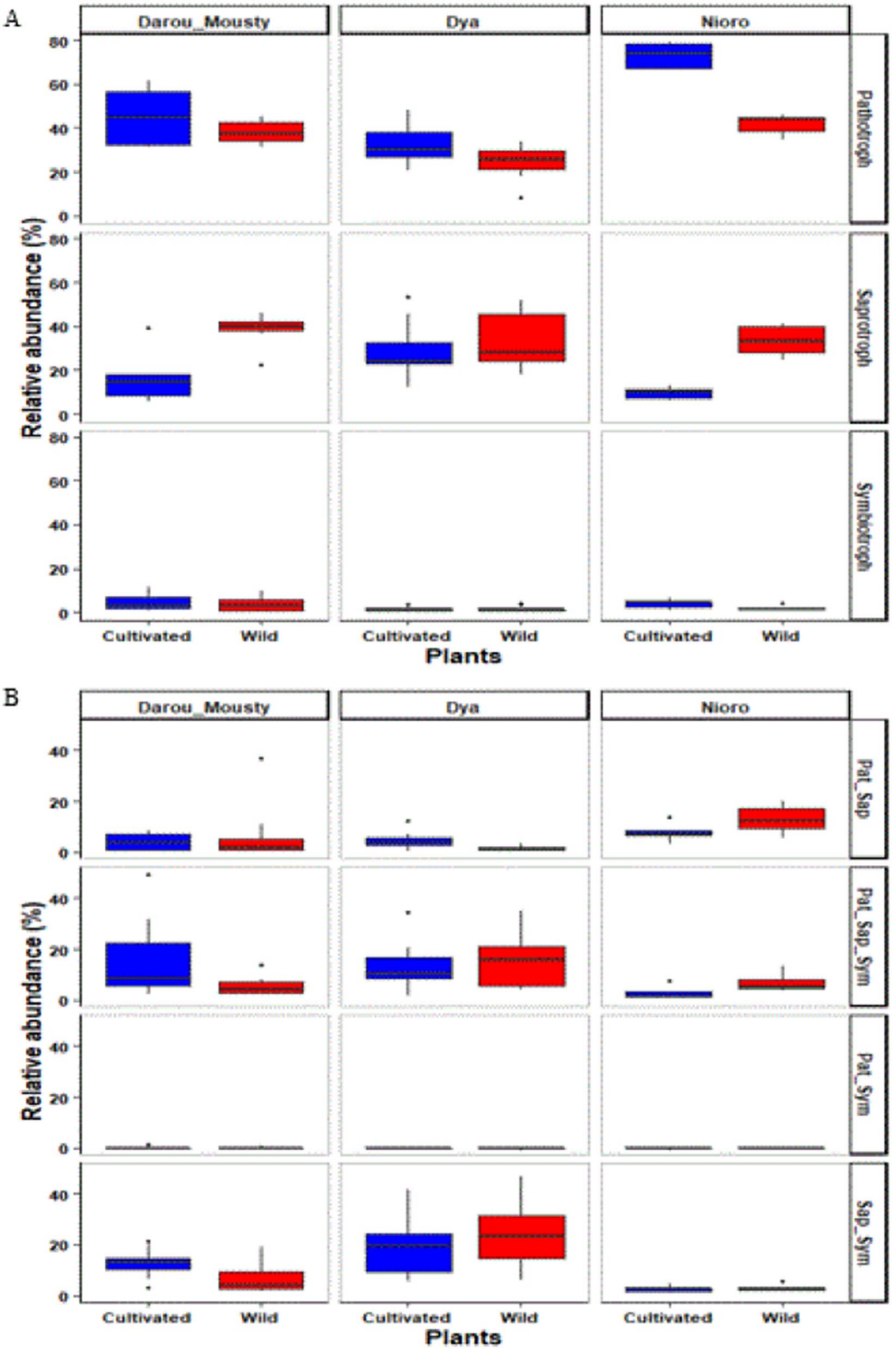
Relative OTU abundance of fungal functional groups associated with cultivated and wild millet across the three sites (Darou-Mousty, Dya and Nioro). The abundances of fungi with one trophic strategy are given in (**A**), and those of fungi with more than one trophic strategy (saprotroph–symbiotroph, pathotroph–symbiotroph, pathotroph–saprotroph, pathotroph–saprotroph–symbiotroph) are in (**B**). In the linear mixed effects (LME) model used to test the effect of plant type and site, plot was included as a random factor (see Table 3).

A total of 4048 out of 5524 OTUs (73%) were assigned to functional guilds using the FUNGuild database. Among them, 2990 OTUs (54%) classified as either pathotroph, saprotroph or symbiotroph (Fig. 4A), while 1058 OTUs (19%) belong to fungi with more than one trophic strategy (Fig. 4B). Putative pathotrophs (38% of relative abundance) and saprotrophs (28% of relative abundance) were the dominant groups in terms of relative abundance in cultivated and wild millet taken together. They were followed by saprotroph–symbiotroph (14% of relative abundance) and pathotroph–saprotroph–symbiotroph (11% of relative abundance).

**Table 3:** Results from ANOVA of the linear mixed effects (LME) model testing the effect of plant type, site and their interaction on the relative abundance of fungal functional groups associated with cultivated and wild millet across the three sites (Darou-Mousty, Dya and Nioro).

Factors	numDF	denDF	F	p	R^2^m	R^2^c
**Pathotrophs**						
Intercept	1	51	193.340	< 0.0001	0.653	0.811
Plants	1	51	40.770	< 0.0001		
Sites	2	3	7.331	0.07		
Plants:sites	2	51	12.901	< 0.0001		
**Saprotrophs**						
Intercept	1	51	114.884	< 0.0001	0.393	0.560
Plants	1	51	31.554	< 0.0001		
Sites	2	3	0.808	0.524		
Plants:sites	2	51	6.710	0.003		
**Symbiotrophs**						
Intercept	1	51	7.335	0.009	0.184	0.740
Plants	1	51	2.350	0.131		
Sites	2	3	0.821	0.520		
Plants:sites	2	51	1.301	0.281		
**Pathotrophs_Saprotrophs**						
Intercept	1	51	11.818	0.001	0.224	0.465
Plants	1	51	0.012	0.913		
Sites	2	3	1.631	0.332		
Plants:sites	2	51	3.379	0.042		
**Pathotrophs_Symbiotrophs**						
Intercept	1	51	17.482	0.0001	0.053	0.053
Plants	1	51	1.610	0.210		
Sites	2	3	0.771	0.537		
Plants:sites	2	51	0.079	0.924		
**Saprotrophs_Symbiotrophs**						
Intercept	1	51	46.181	< 0.0001	0.413	0.557
Plants	1	51	0.023	0.879		
Sites	2	3	5.822	0.093		
Plants:sites	2	51	2.724	0.075		
**Pat_Sap_Sym**						
Intercept	1	51	40.341	< 0.0001	0.189	0.287
Plants	1	51	0.771	0.384		
Sites	2	3	1.744	0.314		
Plants:sites	2	51	3.292	0.045		

R^2^m (marginal r squared) represents the variance explained by the fixed factors, and R^2^c (conditional r squared) represents the variance explained by the both fixed and random factors.

The relative abundance of putative pathotrophs and those of saprotrophs was significantly influenced by plant type and site–plant type interaction (LME, Supplementary Table S6). Putative pathotrophs had a higher relative abundance in cultivated than in wild pearl millet in Dya (*p* = 0.038) and Nioro (*p* < 0.0001) sites. By contrast, saprotrophs had a higher relative abundance in wild than in cultivated millet in Darou-Mousty (*p* = 0.000) and Nioro (*p* = 0.003) sites (Fig. 4A). However, the relative abundance of symbiotrophs was not influenced by plant type, site or their interaction (LME, Supplementary Table S6). Yet, we observed significant site-plant type interaction effects on the relative abundance of pathotroph–saprotroph and those of pathotroph–saprotroph–symbiotroph (Supplementary Table S6, Fig. 4B).

At a finer scale, putative plant pathogens (32% of relative abundance), undefined saprotrophs (17% of relative abundance) and clavicipitaceous endophyte-saprophitic fungi (13% of relative abundance) were the dominant root-associated fungal guilds of cultivated and wild millet taken together. The effects of site and plant type on the 15 most abundant fungal guilds are shown in Supplementary Table S7. The relative abundance of putative plant pathogens was significantly influenced by plant type, site and site—plant type interaction, while those of undefined saprotrophs was only influenced by plant type (Supplementary Table S7). Specifically, undefined saprotroph had a higher relative abundance (*p* = 0.005) in wild than in cultivated millet. Meanwhile, putative plant pathogens had a higher relative abundance in cultivated millet than in their relative wild only in Nioro (*p* = 0.004). In addition, the relative abundance of clavicipitaceous endophyte-saprophitic fungi was not influenced by plant type, site or their interaction (Supplementary Table S7). Altogether, our data indicate that cultivated pearl millet hosts a higher abundance of putative fungal pathogens in semi-arid and semi-humid zones, while wild pearl millet is associated with a higher abundance of fungal saprotrophs in arid and semi-humid zones.

**Figure 5:**
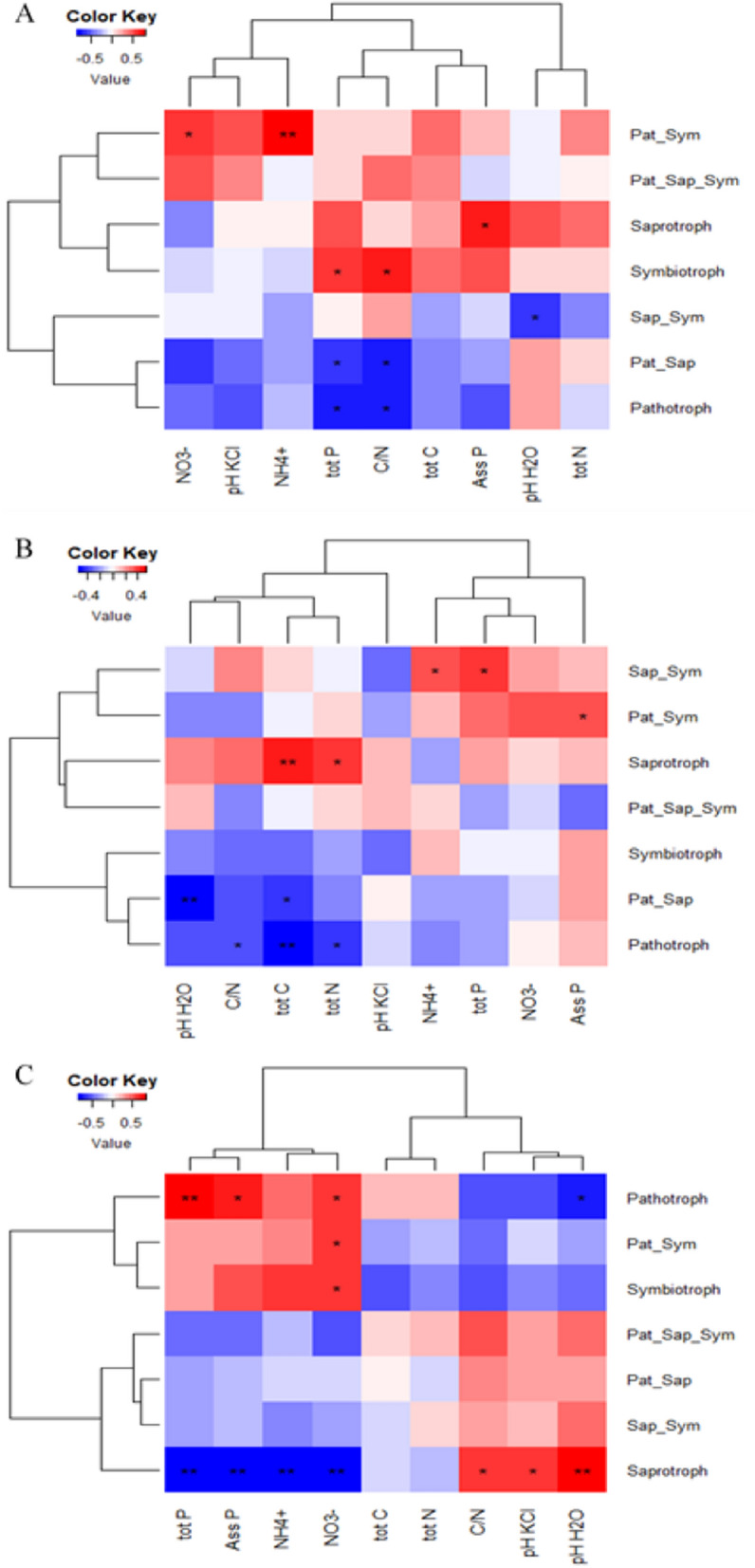
Pearson’s correlation between soil properties and the relative abundance of the 7 trophic mode groups of cultivated and wild millet across the three sites. Stars indicate significant correlation: *p < 0.05, ∗∗*p* < 0.01, ∗∗∗*p* < 0.001. The color key indicates the Pearson correlation coefficient values. For the trophic mode groups, Pat_Sym, Pat_Sap_Sym, Pat_Sap and Sap_Sym refer to pathotroph-symbiotroph, pathotroph-saprotroph-symbiotroph, pathotroph-saprotroph and saprotroph-symbiotroph, respectively. For the soil properties, Ass P and tot P refer to assimilable and total phosphorus respectively. The correlation coefficient r and *p*-values used to produce heatmap figures are given in Supplementary Table S8.

Pearson’s correlations between soil properties and the relative abundance of trophic mode groups revealed contrasting patterns within and across sites (Fig. 5). In Darou-Mousty and Dya for instance, the relative abundance of putative pathotrophs was negatively correlated with total P, C/N, total N and total C, while the relative abundance of saprothrophs was positively correlated with these soil properties. In Nioro, the relative abundance of saprotrophs was positively correlated with pH and C/N, and negatively correlated with total P, assimilable P, NO_3_^−^ and NH_4_^+^, while the relative abundance of pathotrophs followed the opposite pattern for these soil properties (Fig. 5).

### Pearl millet core mycobiome

**Figure 6:**
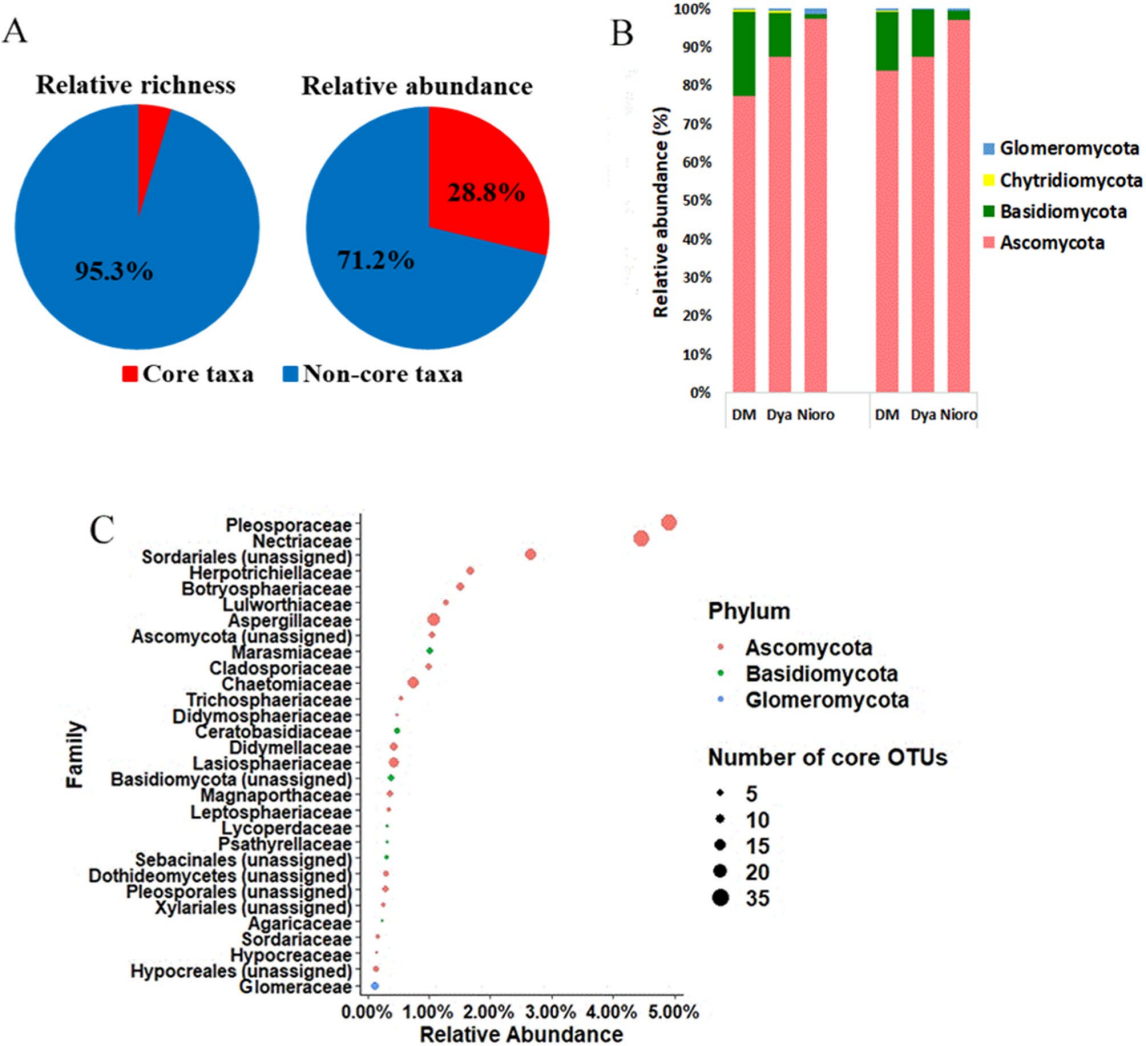
Fungal community composition of the core mycobiome. (**A**) core mycobiome diversity vs all taxa diversity (left) and core microbiome relative abundance vs all taxa (right), (**B**) relative abundance of core mycobiome phyla, (**C**) 30 most abundant family of the core mycobiome.

In order to determine the core mycobiome of pearl millet roots, we used a 75% prevalence threshold to identify the taxa shared between the majority of samples that were collected at sites that were hundreds of kilometers apart and had different soil characteristics. The core mycobiome of millet roots contained 260 OTUs that represented only 4.7% of the observed OTU richness of the entire fungal community. Interestingly, these 260 OTUs accounted for 28.8% of the total sequences of the dataset (Fig. 6A). Of these 260 core OTUs, 91 taxa were found in all of the samples. The 40 most abundant core OTUs (Supplementary Table S9) had relative abundances ranging from 0.17 to 2.04% in the entire dataset. These core taxa belonged to four fungal phyla: Ascomycota (217 OTUs), Basidiomycota (33 OTUs), Chytridiomycota (2 OTUs) and Glomeromycota (8 OTUs). Ascomycota, the most dominant, represented from 63.8 to 97% of the core OTUs with 22 families (Fig. 6C) in pearl millet roots, both in cultivated and wild plants. The most abundant families were *Pleosporaceae* (31 OTUs) and *Nectriaceae* (31 OTUs) with relative abundance of 4.9% and 4.46%, respectively. The second most dominant phylum was Basidiomycota representing from 1.9 to 35.9% of the core OTUs with seven families. In contrast, the phyla Chytridiomycota and Glomeromycota were less represented in the core mycobiome (0.01–1.06% and 0.09–1.28% respectively; Fig. 6B). Some OTUs were classified as OTUs with a very high level of affiliation (Sordariales, Ascomycota, Basidiomycota, Sebacinales, Dothideomycetes, Pleosporales, Xylariales and Hypocreales). All the seven trophic mode groups were represented in the core mycobiome (Supplementary Table S9).

### Co-occurrence patterns in the core mycobiome of cultivated and wild pearl millet

**Figure 7:**
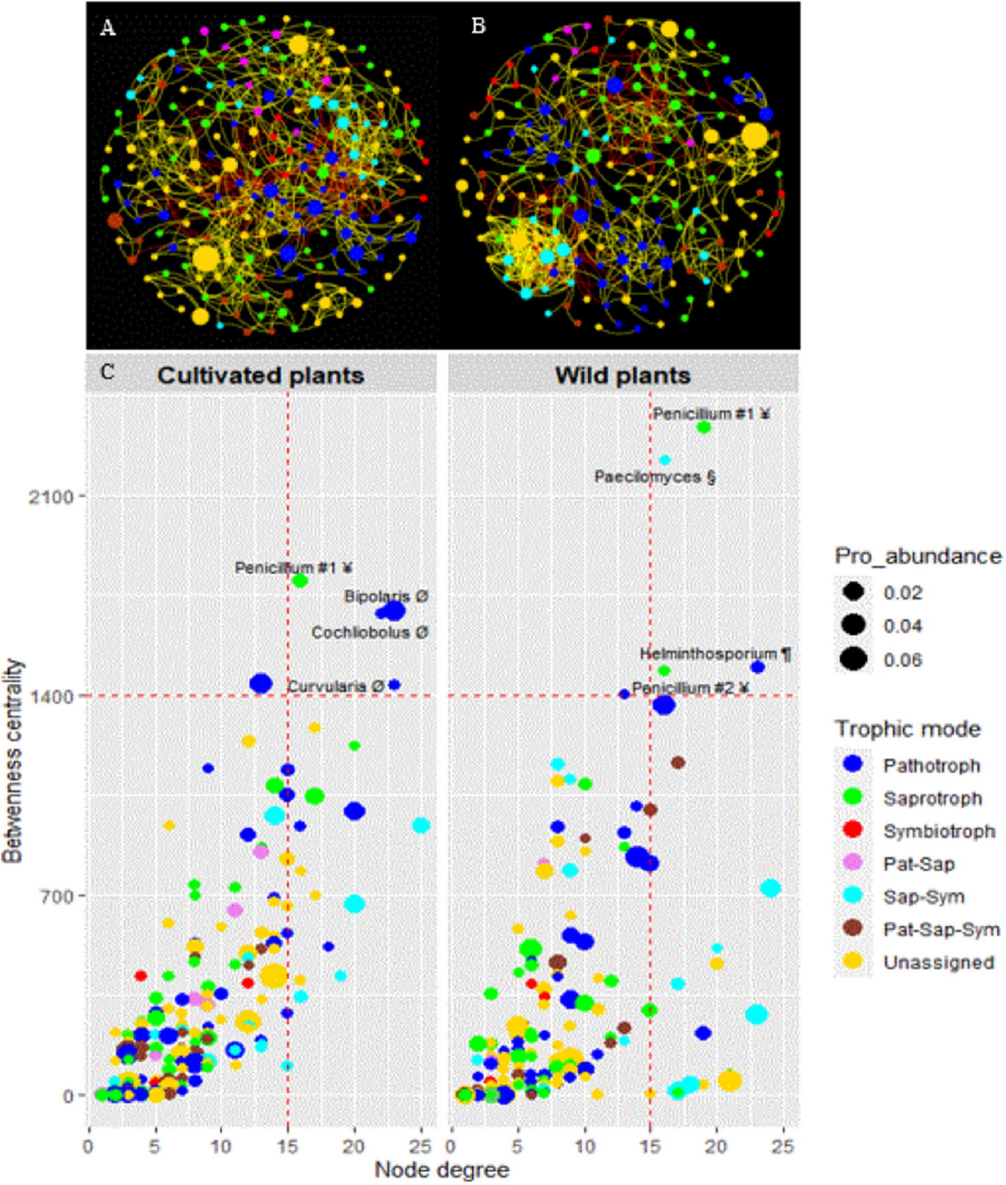
Co-occurrence-based network of the core mycobiome of cultivated (**A**) and wild (**B**) pearl millet. Each node corresponds to an OTU, and edges correspond to either positive (yellow) or negative (red) correlations inferred from OTU abundances. Node size reflects their proportional abundance (Pro_abundance) and their color reflects their trophic mode. Nodes with fungal taxon names represent the potential hub OTUs of cultivated and wild plants inferred from node degree and betweenness centrality values (**C**). Symbols after taxon names indicate their guilds: Ø = plant pathogens, # = dung saprotroph-undefined saprotroph-wood saprotrophs, § = clavicipitaceous endophyte-saprophitic fungi, and ¶ = fungal parasite-plant pathogens. Dashed lines indicate the threshold estimated of the top 2% of degree and betweenness centrality values.

We further analyzed the differences in the core mycobiome of cultivated and wild pearl millet by investigating their taxa co-occurrence patterns using ecological network analysis. The correlation-based network of cultivated plants contained 214 nodes and 807 edges, while that of the wild plants had 195 nodes and 680 edges (Fig. 7A,B). The networks of cultivated and wild plants had 84.51% and 84.12% of strong positive correlations, respectively. Except for average degree, all estimated network-level topological features (average path length, network diameter, graph density, modularity and clustering coefficient) were higher in wild than in cultivated pearl millet (Supplementary Table S10). The betweenness centrality and eigenvector centrality were higher in the network of cultivated plants compared to that of wild plants, while node degree and closeness centrality did not differ significantly between the two networks (Supplementary Fig. S1). In addition, we identified four potential hubs belonging to three genera which include plant pathogen (*Bipolaris*, *Cochliobolus* and *Curvularia*) and one dung saprotroph-undefined saprotroph-wood saprotroph (*Penicillium* #1) in the ecological network of cultivated pearl millet. By contrast, four potential hubs belonging to two dung saprotroph-undefined saprotroph-wood saprotroph (*Penicillium* #1 and *Penicillium* #2), one clavicipitaceous endophyte-saprophitic fungi (*Paecilomyces*) and one fungal parasite–plant pathogen (*Helminthosporium*) were identified in the ecological network of wild pearl millet (Fig. 7C). The two ecological networks shared only one potential hub (*Penicillium* #1). Furthermore, these seven potential hub taxa belonged to three trophic mode groups including saprotroph, pathotroph and saprotroph–symbiotroph (Fig. 7C).

## Discussion

Here, we analyzed the root mycobiome of cultivated and wild pearl millet and the environmental factors structuring its diversity across three agro-ecological zones in Senegal. Our results show that root fungal communities were mainly structured by sites and plant type. Soil pH and phosphorus were the main factors explaining the site effect on pearl millet fungal communities as reported in previous studies^27–31^. We observed that, in each site, the relationship between the relative abundance of putative pathotrophs and soil properties (especially total P, C/N, total N and total C) and the relationship between the relative abundance of saprotrophs and those soil properties followed opposite trends. On the other hand, contrasting patterns were observed across sites. This finding contrasts with others that showed that abundance of pathogenic fungi followed the same trend as that of saprotrophic fungi^32,33^. Although we relate the site effect observed on the relative abundance of most guilds and trophic mode groups to differences in soil properties, we cannot rule out the contribution of other factors such as host plant density, temperature, rainfall and moisture^34–37^. We acknowledge that the fungal trophic modes were identified by FUNGuild which is based on fungal taxonomy, and therefore studies about the fungal lifestyles and trophic modes are needed to better understand the factors influencing the contrasting patterns we observed. In addition, the differences in soil properties observed between and within sites could be related to different factors including the quantity and type of inputs used, cropping system, land use history and plant functional trait effects^38–41^.

We observed a higher fungal diversity in cultivated compared to wild pearl millet. We speculate that during pearl millet domestication, genotypic and phenotypic changes resulting from selection and demographic bottlenecks could have affected root traits related to nutrient foraging and plant–microbe interaction in response to resource availability and heterogeneity^42–44^. In addition, we suggest that additional adaptive changes in the root morphological traits and quantitative and/or qualitative root exudate composition of domesticated pearl millet due to constant clogging effects over time may have facilitated colonization by a broader range of fungi^45–48^. Interestingly, we detected higher relative abundance of putative pathotrophs, especially potential plant pathogens, in cultivated pearl millet than in wild pearl millet in semi-arid and semi-humid zones, while having higher relative abundance of saprotrophs in wild pearl millet in arid and semi-humid zones. This suggests that cultivated pearl millet plants were more colonized by pathotrophs than wild relatives, while the latter provided a more appropriate ecological niche for saprotrophs across the three studied agro-ecological zones. Besides genotypic and phenotypic differences between cultivated millet and wild relatives, resource demand, exudate and litter quality, interaction with other microbes, agriculture practices, and moisture may account for this observation^9,42,49^. Although further studies are needed to better understand factors behind the selection of fungal communities by wild and cultivated pearl millet, the fact that the relative abundance of putative pathotrophs was lower in wild millet suggests that it could be a potential resource of genetic resistance to root pathogens^50,51^. On the other hand, although we believe that our results reflected intrinsic differences between cultivated and wild pearl millet in terms of diversity of their root-associated fungi, they should be interpreted cautiously since our sequencing depth was insufficient to cover the entire fungal diversity in both cultivated and wild pearl millet (Supplementary Fig. S2).

We hypothesized that wild pearl millet could serve as a reservoir of endophytes for cultivated pearl millet. We therefore characterized a pearl millet core mycobiome across three contrasted regions. Interestingly, it was composed of diverse groups including 260 taxa that accounted for almost 28.8% of total abundance of fungal community in our dataset indicating that these taxa are both prevalent and abundant on millet roots. In our study, Ascomycota were dominant in the core mycobiome. Basidiomycota was the second most dominant phylum, and Glomeromycota was the least represented in the core mycobiome. The lower proportion of Glomeromycota could be related to the low levels of pearl millet root colonization by AMF (root length colonization ≤ 5%^52^). The ecological network analysis we performed revealed a predominance of strong positive correlations between fungal OTUs in both cultivated and wild millet (84.51% and 84.12%, respectively), suggesting a potential for extensive cooperative interactions or sharing similar ecological niches between most fungal taxa in pearl millet roots^53,54^. Our results also showed that wild plants had lower betweenness centrality, but higher network-level topological features (e.g. network diameter, graph density, modularity and clustering coefficient) compared to cultivated plants, indicating a more complex and connected network^55,56^. This higher network complexity in wild pearl millet may result in a more stable mycobiome that could contribute to higher plant resilience to environmental perturbations^57,58^. On the other hand, plant genotype and/or agriculture practices may account for the lower network complexity in cultivated pearl millet^59,60^.

We identified four different potential hub taxa in the core mycobiome of cultivated (*Bipolaris*, *Cochliobolus*, *Curvularia* and *Penicillium* #1) and wild (*Helminthosporium*, *Paecilomyces*, *Penicillium* #1 and *Penicillium* #2) plants. These hub taxa may act as keystone taxa^56,61^ in the mycobiome with a strong structuring or recruiting role in the community. Surprisingly, the cultivated millet and wild relatives had dissimilar groups of potential hub taxa (only one was shared), suggesting that they may develop dissimilar interactions with their mycobiome for biotic and abiotic stress tolerance^48,62^. Similarly, analyses of seed microbiome of domesticated and wild rice showed that they had different hub OTUs^61^. In our study, two potential hub taxa including the shared one, belonged to *Penicillium,* that was also identified as hub taxon in the seed microbiome of wild rice^61^. Besides its role in nutrient cycling through the decomposition of organic matter, *Penicillium* is known to solubilize phosphate^63–65^ and to excrete antimicrobial substances^66,67^. In this respect, Murali and Amruthesh^68^ have shown that *Penicillium oxalicum,* significantly reduced the downy mildew disease and enhanced plant growth in pearl millet. We also identified *Paecilomyces*, a clavicipitaceous endophyte-saprophitic fungus, as a potential hub taxon in the core mycobiome of wild pearl millet. *Paecilomyces* fungi are known to produce bioactive metabolites and therefore have been used as plant growth-promoting fungi for horticultural crops^69^. Moreover, it has been reported that extracts of some *Paecilomyces* strains combined with urea or phosphate increased the yield of rice and maize^70,71^. Most of clavicipitaceous fungi are members of dark septate endophyte (DSE) which can be in some cases more frequent than mycorrhizal fungi^5,72,73^. They play a significant ecological and physiological role in different ecosystems by impacting plant growth and nutrition. In the pearl millet core mycobiome, we identified at least six families (*Cladosporiaceae*, *Herpotrichiellaceae*, *Didymellaceae*, *Didymosphaeriaceae*, *Leptosphaeriaceae*, *Pleosporaceae* and *Trichosphaeriaceae*) and two unassigned taxa at family level (Sordariales and Xylariales), containing DSE fungi. The high relative abundance and ubiquity of members of DSE suggest a mutualistic lifestyle that might compensate for low levels of AMF colonization in pearl millet^74^. Interestingly, the other potential hub taxa identified in this study and assigned as plant pathogens (*Bipolaris*, *Cochliobolus* and *Curvularia*) and fungal parasite-plant pathogen (*Helminthosporium*), have also been reported to be phosphate-solubilizing fungi^75–79^. Their impact on the fitness of cultivated and wild pearl millet remains to be elucidated.

In conclusion, this study allowed us to assess the factors influencing fungal communities associated with wild and cultivated pearl millet roots. We found that soil pH and available phosphorus were the main factors explaining the effect of sites on fungal communities. On the other hand, we observed a higher fungal diversity in cultivated compared to wild pearl millet. Interestingly, our data indicate that cultivated pearl millet hosts a higher relative abundance of potential fungal pathogens while wild pearl millet is associated with a higher relative abundance of fungal saprotrophs. In addition, the cultivated millet and wild relatives had dissimilar groups of hub taxa (only one out of eight, was shared), suggesting that they may develop dissimilar sophisticated dialogues with their mycobiome for biotic and abiotic stress tolerance. Future research should focus on these core and hub taxa, which are likely to play an important role in pearl millet fitness and will allow the development of microbiome engineering approaches for agriculture.

## Methods

### Site description

The study was conducted in Senegal, West Africa, in the North–South region of the groundnut basin (13° 45′ N, 15° 47′ W) at 18 m above sea level, specifically in the district of Darou-Mousty (15° 02′ 31″ N, 16° 02′ 53″ W) with two plots, Dya (14° 23′ 60″ N, 16° 10′ W, 11 m altitude) with three plots and Nioro (13° 45′ N, 15° 48′ W, 19 m altitude) with one plot (Supplementary Fig. S3). The two main crops grown in these areas are millet (*P. glaucum*) and groundnuts (*Arachis hypogaea*). The mean annual precipitation is 750 mm and mainly comes between July and September, and the annual average temperature is 30 °C^80^. The soil is a Deck-Dior^81^ silty-sand fine sandy, mixed haplic ferric lixisol^82^, a leached ferruginous tropical soil. Top soil (0–10 cm) has a sand content > 90%, organic matter and total nitrogen content of 0.52 and 0.03% respectively, total P content of 70 mg kg^−1^ and an average pH (water) of 6.2^83^. The sites were chosen on the basis of soil homogeneity (Dior type) following a gradient of rainfall from North to South.

### Experimental design and soil/root sampling

The experiment was conducted during the 2016 rainy season, with a mean precipitation of 476, 518 and 911 mm in Darou-Mousty (arid zone), Dya (semi-arid zone) and Nioro (semi-humid zone), respectively. Field trials were conducted in collaboration with local farmers’ associations and sampling was performed with the help of farmers. Permission to collect plants and soils was obtained from farmers before settling the trials. Trials did not involve endangered or protected species and complied with relevant national and international guidelines and legislation. Seeds from a single pearl millet variety (Souna 3) were provided to all farmers participating to the trials on all sites in order to limit plant genotype effect. The farmers followed their traditional agricultural practices (fertilization or not, previous crops, etc.) that were recorded as well as the cropping history and the vegetation around (Supplementary Table S11).

At the end of the grain-filling stage, soil and root samples were taken. From each cultivated plot, a set of five replicates was collected. Each biological replicate for sequencing analysis came from the roots of five plants harvested from each cultivated plot. Sampled plants were located 10 m from each other to cover the whole plot. Soils and roots of wild relatives were collected within or around the cultivated plots. For each individual plant, approximately 500 g of soil influenced by the roots was collected at a depth of 0–20 cm. A total of 60 root samples and 60 soil samples were collected: two plant types (cultivated and wild), six plots and five replicates per plot. Soils and roots were placed in plastic bags in ice and transported to the laboratory where they were stored at 4 °C for 24 h before processing. Total DNA extraction was only done from the roots while the soils samples were used for physicochemical analyses. Wild pearl millet is morphologically dissimilar to cultivated pearl millet. Supplementary Figure S4 shows the differences between cultivated and wild pearl millet.

### Soil chemical analysis

Soil pH was determined with a soil-to-water ratio of 1: 2.5. Total carbon (C) and total nitrogen (N) contents were quantified using Elemental Analyzer (Flash EA 1112 series, ThermoFinnigan, France). Soil nitrate (NO_3_^−^) and ammonium (NH_4_^+^) were extracted with 2 M KCl and were quantified by Bran + Luebbe GmbH AutoAnalyzer 3. Soil available phosphorus (AP) was extracted using sodium bicarbonate and then measured by the molybdenum-blue method^84^. The P concentration was determined after dry mineralization by inductively coupled plasma atomic emission spectrometry (ICP-AES^85^).

### DNA-metabarcoding

Root samples were cleaned of soil particles by manual agitation and about 100 mg of fine roots from each replicate were collected and ground with 270 mg of Fontainebleau sand (minimum quantity allowing the crushing of 100 mg of roots) with a mortar and pestle. 185 mg of the obtained grind (corresponding to approximately 50 mg of root material) was used for DNA extraction. No specific treatment was applied to the roots, so the mycobiome considered here included both fungi inhabiting the root surface and root endosphere of pearl millet. Total DNA was extracted using the DNeasy Plant Mini Kit (Qiagen^®^) according to the manufacturer's instructions. A total of 60 DNA samples (30 cultivated and 30 wild) were obtained. The concentration and purity of isolated DNA were determined using a Nanodrop ND-2000 UV–VIS spectrophotometer (NanoDrop Technologies, Wilmington) and DNA samples were stored at − 20 °C before sequencing.

DNA amplification and sequencing of fungal rDNA were performed at MR DNA (www.mrdnalab.com, Shallowater, TX, USA). Briefly, the ITS1 region of fungal rDNA was amplified by PCR using 5 µL of Q5 Reaction Buffer (5×), 5 µL of Q5 GC high Enhancer (5×), 1 µL (10 µM) of ITS1F primer (5′-CTTGGTCATTTAGAGGAAGTAA-3′;^86^), 1 µL (10 µM) of ITS2 primer (5′-GCTGCGTTCTTCATCGATGC-3′;^87^), 2 µL of dNTP (2.5 µM), 1 µL of DNA template (20 ng µL^−1^), 0.25 µL of Q5 Polymerase (5U µL^−1^) and 9.75 µL of ddH2O to a final volume of 25 µL. PCR was performed in triplicate using the following conditions: 5 min at 98 °C for initial denaturing, followed by 27 cycles of 98 °C for 30 s, primer annealing at 56 °C for 30 s and extension at 72 °C for 30 s, followed by a final extension for 5 min at 72 °C.

After amplification, the quality and relative concentration of the amplicons were checked by migration on 2% agarose gel. Multiple replicates were pooled together in equal proportions based on their molecular weight and DNA concentrations. Pooled DNA samples were purified using calibrated Ampure XP beads. Then the pooled and purified amplicons were used to prepare DNA libraries following Illumina Truseq DNA library preparation protocol. Sequencing was performed on a MiSeq Illumina platform (2 × 300) following the manufacturer’s guidelines.

### Sequence analysis

First, raw Illumina MiSeq paired-end reads were assembled using MR DNA pipeline for ITS1 region fungal libraries. Subsequently, sequences were demultiplexed and formatted for processing using a Phython script (http://drive5.com/usearch/manual/uparse_pipeline.html). Next, fungal sequences were separately quality-filtered and clustered into operational taxonomic units (OTUs) using UPARSE pipeline and UPARSE algorithm^88^. Briefly, sequences were quality-filtered allowing a maximum e value of 0.5. Subsequently, reads were trimmed to 240-bp (base pairs) length as well as dereplicated and sorted by abundance, removing singletons (sequences which appeared once) prior OTU determination at 97% sequence similarity threshold. Then, chimeric sequences were detected and removed using UCHIME^89^ and Gold database as reference. Finally, reads from the entire dataset were mapped back to the representative fungal databases to generate one OTU table. The taxonomic affiliation of each OTU was obtained using the UNITE database (version 7.2, https://unite.ut.ee,^90^). OTUs were then assigned to functional groups using the FUNGuild database (https://github.com/UMNFuN/FUNGuild^15^). We only accepted the guild assignment that confidence rankings were “highly probable” or “probable”. For taxa with more than one trophic strategy, we subsequently used manual evaluation as recommended by Nilsson et al.^16^. The raw sequence data has been deposited in figshare (https://figshare.com/articles/Pearl_Millet_Fungus/11277950).

### Data analyses

The number of sequences per sample varied from 25,556 to 154,517. Because the library sizes were very uneven, the data were normalized to the same number of counts per sample (25,556 sequences) using the “rarefy” function in the “Vegan” package^91^ in R (v 4.1.0; h ttps://cran.r-project.org) as recommended by Weiss et al.^92^. Species richness, Shannon and Simpson diversity indices were used to assess α-diversity of fungal communities associated with cultivated and wild pearl millet. Before testing the differences between the means, we first visualized the distribution of the data using a histogram to determine the distribution of errors. Then, the Shapiro test was used to check normality followed by the Bartlett test to check for homoscedasticity of variances. As normality was verified, the data were subjected to a linear mixed-effects (LME) model fit by restricted maximum likelihood (REML) with plant type, site, and their interaction as fixed factors and plot as random factor using the “lme” function in the “nlme” package^93^. The significance of fixed effects was assessed using the “anova.lme” function. If there was a significant effect of the site or the site-plant type interaction, pairwise comparisons were conducted using the “emmeans” package with Tukey’s adjusted p values^94^. The variance explained by the fixed factors (marginal R^2^) and those explained by both the fixed and random factors (conditional R^2^) were calculated with the “r.squaredGLMM” function in the “MuMIn” package^95^. Differences in the community structure between samples (β-diversity) were visualized using a non-metric multidimensional scaling (NMDS) based on Bray–Curtis distances calculated from the "metaMDS" function implemented in the "Vegan" package^92^. The “betadisper” function in “Vegan” was used to compare group dispersions. The "envfit" function was used to determine the relationships between community structure and soil properties. The PERMANOVA test (non-parametric Permutational Multivariate Analysis of Variance) was used to test significant differences in the structure of fungal communities by the "adonis" function^96^.

Differentially abundant fungal OTUs between plant types (cultivated vs wild pearl millet) were identified in each site by the “DESeq2” package^97^ using the default values of the "Test (Wald)" and "FitType (parametric)" options of the function. The differential abundance calculation is based on a modeling of the OTU distribution by a negative binomial law. The adjusted *p* value cutoff was set to alpha = 0.01, and the list of OTUs declared differentially abundant was extracted. Considering only the assigned OTUs using FUNGuild, we calculated the relative abundance of each guild and trophic mode group, and a LME model fit by REML was used to assess the effects of plant type, site, and their interaction. The model included plant type, site, and their interaction as fixed factors and plot as random factor. The significance of fixed effects was assessed using the “anova.lme” function. If there was a significant effect of the site or the site-plant type interaction, pairwise comparisons were conducted as described above. The variance explained by the fixed factors and those explained by both fixed and random factors were calculated with the “r.squaredGLMM” function. For each site, we used Pearson’s correlation coefficients to determine the relationship between soil properties and the relative abundance of each trophic mode group using the "rcorr" function in the "Hmisc" package^98^, and a heatmap was then built using the "heatmap.2" function in the "gplots" package^99^.

We characterized the core fungal microbiome (mycobiome) of pearl millet roots on a 75% prevalence threshold using the "microbiome" package^100^ to identify highly prevalent taxa on pearl millet roots that are present in the majority of samples (across all sites and plant types). We then constructed co-occurrence networks to infer interactions among OTUs in the core mycobiome of cultivated plants and wild plants, based on Spearman’s correlation inferred from OTU abundances. We considered only positive correlations (with r > 0.6) and negative correlations (with r < − 0.6) associated with FDR-adjusted *p* values < 0.01^55,101^. Gephi software (v0.9.2; https://gephi.org) was used to visualize the ecological networks and estimate node-level topological features (degree, betweenness centrality, closeness centrality and eigenvector centrality) and network-level topological features (average degree, average path length, network diameter, graph density, modularity and clustering coefficient) for the cultivated and wild plants^55^. In each network, nodes correspond to OTUs, and edges correspond to strong correlations inferred from OTU abundances. For each ecological network, the OTUs belonging to the top 2% of degree and betweenness centrality were identified as potential hub OTUs^61^. We then used the Wilcoxon test to compare the estimated node-level topological features between cultivated and wild plants, and graphics were made using the ‘ggplot’ package^102^.

## Supplementary Information

Supplementary Information.

Supplementary Table S4.

Supplementary Table S5.

Supplementary Table S8.

Supplementary Table S9.
