# Author Correction: Cultivated and wild pearl millet display contrasting patterns of abundance and co-occurrence in their root mycobiome

**DOI:** 10.1038/s41598-022-05899-0

**Published:** 2022-01-25

**Authors:** Marie-Thérèse Mofini, Abdala G. Diedhiou, Marie Simonin, Donald Tchouomo Dondjou, Sarah Pignoly, Cheikh Ndiaye, Doohong Min, Yves Vigouroux, Laurent Laplaze, Aboubacry Kane

**Affiliations:** 1 Département de Biologie Végétale, Faculté des Sciences et Techniques, Université Cheikh Anta Diop (UCAD), BP 5005, Dakar Fann, Senegal; 2 Laboratoire Mixte International Adaptation des Plantes et Microorganismes associés aux Stress Environnementaux (LAPSE), Centre de recherche de Bel-Air, Dakar, Sénégal; 3 Laboratoire Commun de Microbiologie (LCM), Centre de Recherche de Bel-Air, Dakar, Sénégal; 4 Centre d’Excellence Africain en Agriculture pour la Sécurité Alimentaire et Nutritionnelle (CEA-AGRISAN), UCAD, Dakar, Sénégal; 5 Centre d’Etude Régional pour l’Amélioration de l’Adaptation à la Sécheresse (CERAAS), Institut Sénégalais de Recherches Agricoles (ISRA), Route de Khombole, Thiès, Sénégal; 6 IPME, IRD, Cirad, Université de Montpellier, Montpellier, France; 7 DIADE, Université de Montpellier, IRD, Cirad, 911 Avenue Agropolis, 34394 Montpellier cedex 5, France; 8 Department of Agronomy, Kansas State University, Manhattan, KS USA; 9 Present Address: Université d’Angers, Institut Agro, INRAE, IRHS, SFR 4207 QuaSaV, 49000 Angers, France

Correction to: *Scientific Reports* 10.1038/s41598-021-04097-8, published online 07 January 2022

The original version of this Article contained errors in Supplementary Information 1, where Supplementary Figure 1 was incorrectly given as Supplementary Figure 2. In addition, Supplementary Figure 2 was omitted. The original Supplementary Information 1 file is provided below.

Additionally, the Figure 5 legend was incomplete.

“Pearson’s correlation between soil properties and the relative abundance of the 7 trophic mode groups of cultivated and wild millet across the three sites. Stars indicate significant correlation: *p < 0.05, ∗∗p < 0.01, ∗∗∗p < 0.001.”

now reads:

“Pearson’s correlation between soil properties and the relative abundance of the 7 trophic mode groups of cultivated and wild millet across the three sites. Stars indicate significant correlation: *p < 0.05, ∗∗p < 0.01, ∗∗∗p < 0.001. The color key indicates the Pearson correlation coefficient values. For the trophic mode groups, Pat_Sym, Pat_Sap_Sym, Pat_Sap and Sap_Sym refer to pathotroph-symbiotroph, pathotroph-saprotroph-symbiotroph, pathotroph-saprotroph and saprotroph-symbiotroph, respectively. For the soil properties, Ass P and tot P refer to assimilable and total phosphorus respectively. The correlation coefficient r and p-values used to produce heatmap figures are given in Supplementary Table S8.”

The original Article and accompanying Supplementary Information file have been corrected.

## Supplementary Information

Supplementary Information.

