# Supplementary material for: Author Correction: Cultivated and wild pearl millet display contrasting patterns of abundance and co-occurrence in their root mycobiome
Source: Sci Rep. 2022 Jan 25;12:1639. doi: 10.1038/s41598-022-05899-0 (PMC8789787; doi:10.1038/s41598-022-05899-0)
Supplement: Supplementary file 1 — Supplementary Information. [file 41598_2022_5899_MOESM1_ESM.pdf]

Supplementary information

Cultivated and Wild Pearl Millet Display Contrasting Patterns of Abundance and Co-occurrence in their root mycobiome

Marie-Thérèse Mofini, Abdala G. Diedhiou, Marie Simonin, Donald Tchouomo Dondjou, Sarah Pignoly, Cheikh Ndiaye, Doohong Min, Yves Vigouroux, Laurent Laplaze, and Aboubacry Kane

Supplementary Table S1: Composition of soils sampled from the three agro-ecological zones of Darou-Mousty (arid zone), Dya (semi-arid zone) and Nioro (semi-humid zone)

	**Darou Mousty**	**Dya**	**Nioro**	**P value**
**pH H_2O**	6.65 ± 0.17 a	6.37 ± 0.32 b	5.51 ± 0.33 c	5.15e-11
**pH KCl**	5.79 ± 0.11 a	5.49 ± 0.32 b	4.54 ± 0.10 c	2.61e-14
**NO_3^-**	0.93 ± 1.23 a	0.73 ± 1.07 a	1.46 ± 1.02 a	0.199
**NH_4^+**	7.71 ± 1.79 b	7.43 ± 3.81 b	12.16 ± 4.23 a	0.003
**N tot**	0.04 ± 0.01 a	0.03 ± 0.01 ab	0.02 ± 0.01 b	0.016
**C tot**	0.39 ± 0.05 a	0.35 ± 0.14 a	0.31 ± 0.04 a	0.237
**C/N**	10.13 ± 0.75 b	10.66 ± 0.62 b	11.58 ± 0.38 a	1.29e-05
**P tot**	89.15 ± 31.49 ab	169.74 ± 116.88 a	71.38 ± 15.65 b	0.006
**P assim**	5.44 ± 1.90 a	5.08 ± 2.63 a	6.42 ± 3.54 a	0.406

Means in the same line followed by the same letter are not significantly different ($P < 0.05$) according to Tukey's HSD. N tot: total nitrogen; C tot: total carbon; C/N: carbon to nitrogen ratio; P tot: total phosphorus; P assim: assimilable phosphorus.

Supplementary Table S2: Results from ANOVA of the linear mixed effects (LME) model testing the effect of plant type, site and their interaction on species richness, Shannon and Simpson diversity indexes of the fungal communities associated with cultivated and wild Pearl millet. R^2m (marginal r squared) represents the variance explained by the fixed factors, R^2c (conditional r squared) the variance explained by the both fixed and random factors, numDF the numerator degree of freedom and denDF the denominator degree of freedom.

Factors	numDF	denDF	F	P	R^2m	R^2c
Richness						
Intercept	1	51	6450.426	<0.0001	0.310	0.310
Plants	1	51	19.153	0.000		
Sites	2	3	0.936	0.483		
Plants:Sites	2	51	2.736	0.074		
Shannon						
Intercept	1	51	5988.246	<0.0001	0.216	0.258
Plants	1	51	14.182	0.000		
Sites	2	3	0.755	0.542		
Plants:Sites	2	51	0.321	0.727		
Simpson						
Intercept	1	51	32490.240	<0.0001	0.162	0.209
Plants	1	51	7.470	0.009		
Sites	2	3	1.100	0.439		
Plants:Sites	2	51	0.530	0.591		

Supplementary Table S3: Summary of permutational analysis of variance (PERMANOVA) based on Bray-Curtis distance to test the effects of site and plant type on the structure of fungal communities associated to pearl millet roots.

**Factors**	***df***	**SS**	**MS**	**F.Model**	**R^2**	***p*-value**
**Sites**	2	3.1885	1.59425	7.9021	0.21097	0.001
**Plants**	1	0.4361	0.43606	2.1614	0.02885	0.001
**Sites*Plants**	2	0.5947	0.29737	1.4739	0.03935	0.005
**Residuals**	54	10.8945	0.20175	0.72083		
**Total**	59	15.1138	1.00000			

df = degrees of freedom; SS = sum of squares; MS = mean sum of squares; *F* model = *F* statistics; R^2= partial R-squared, based on 999 permutations.

Supplementary Table S4: Relation between soil properties and fungal community structure. Correlations community structure with environmental variables used Envfit function.

Variable	NMDS1	NMDS2	R^2	P-value
**pH H_2O**	-0.32	-0.94	0.31	0.001
**pH KCl**	-0.49	-0.86	0.36	0.001
**NO_3^-**	-0.41	0.91	0.07	0.16
**NH_4^+**	-0.27	0.96	0.09	0.10
**Total N**	-0.66	-0.74	0.05	0.28
**Total C**	-0.67	-0.73	0.03	0.40
**C/N ratio**	0.99	0.12	0.05	0.24
**Total P**	-0.37	-0.92	0.17	0.01
**Assimilable P**	-0.66	0.75	0.13	0.03

Supplementary Table S6: Results from ANOVA of the linear mixed effects (LME) model testing the effect of plant type, site and their interaction on the relative abundance of fungal functional groups associated with cultivated and wild millet across the three sites (Darou-Mousty, Dya and Nioro). R^2m (marginal r squared) represents the variance explained by the fixed factors, R^2c (conditional r squared) the variance explained by the both fixed and random factors, numDF the numerator degree of freedom and denDF the denominator degree of freedom.

**Factors**	**numDF**	**denDF**	**F**	**P**	**R^2m**	**R^2c**
Pathotrophs						
Intercept	1	51	193.340	<0.0001	0.653	0.811
Plants	1	51	40.770	<0.0001		
Sites	2	3	7.331	0.07		
Plants:Sites	2	51	12.901	<0.0001		
Saprotrophs						
Intercept	1	51	114.884	<0.0001	0.393	0.560
Plants	1	51	31.554	<0.0001		
Sites	2	3	0.808	0.524		
Plants:Sites	2	51	6.710	0.003		
Symbiotrophs						
Intercept	1	51	7.335	0.009	0.184	0.740
Plants	1	51	2.350	0.131		
Sites	2	3	0.821	0.520		
Plants:Sites	2	51	1.301	0.281		
Pathotrophs_Saprotrophs						
Intercept	1	51	11.818	0.001	0.224	0.465

Plants	1	51	0.012	0.913		
Sites	2	3	1.631	0.332		
Plants:Sites	2	51	3.379	0.042		
Pathotrophs_Symbiotrophs						
Intercept	1	51	17.482	0.0001	0.053	0.053
Plants	1	51	1.610	0.210		
Sites	2	3	0.771	0.537		
Plants:Sites	2	51	0.079	0.924		
Saprotrophs_Symbiotrophs						
Intercept	1	51	46.181	<0.0001	0.413	0.557
Plants	1	51	0.023	0.879		
Sites	2	3	5.822	0.093		
Plants:Sites	2	51	2.724	0.075		
Pat_Sap_Sym						
Intercept	1	51	40.341	<0.0001	0.189	0.287
Plants	1	51	0.771	0.384		
Sites	2	3	1.744	0.314		
Plants:Sites	2	51	3.292	0.045		

Supplementary Table S7: Site and plant type effects on the 15 most abundant fungal guilds associated with Pearl millet**.** In the linear mixed effects (LME) model used to test the effect of plant type and site, plot was included as a random factor.

**Fungal guilds**	**Darou Mousty**		**Dya**		**Nioro**		**Factors tested**		
	**Cultivated (Mean±SEM)**	**Wild (Mean±SEM)**	**Cultivated (Mean±SEM)**	**Wild (Mean±SEM)**	**Cultivated (Mean±SEM)**	**Wild (Mean±SEM)**	**Plants**	**Sites**	**Plants x Sites**
Plant pathogen	36.60 ± 16.49	27.93 ± 7.63	28.10 ± 13.65	20.97 ± 12.99	67.99 ± 5.18	37.67 ± 3.75	**	*	*
Undefined saprotroph	9.10 ± 7.87	32.03 ± 7.24	16.91 ± 8.81	21.69 ± 13.44	5.50 ± 2.16	26.86 ± 6.71	**	NS	NS
Clavicipitaceous endophyte-Undefined/Wood saprotroph	9.67 ± 4.87	7.55 ± 5.89	16.81 ± 11.40	23.53 ± 13.28	1.53 ± 0.47	1.66 ± 0.78	NS	NS	NS
Fungal parasite-Plant pathogen	7.97 ± 5.88	7.51 ± 6.26	3.77 ± 2.95	2.70 ± 1.99	3.16 ± 1.44	0.42 ± 0.36	NS	*	NS
Endophyte-Plant pathogen-Undefined saprotroph	9.92 ± 18.80	1.26 ± 1.82	3.64 ± 7.61	2.58 ± 4.56	0.19 ± 0.02	0.32 ± 0.27	NS	NS	NS
Dung saprotroph-Undefined saprotroph-Wood saprotroph	0.38 ± 0.25	0.61 ± 0.65	2.13 ± 2.89	7.65 ± 12.77	1.83 ± 0.82	4.43 ± 1.91	NS	NS	NS
Animal patho-Endo-Plant/Soil/Undefined/Wood saprotroph	0.95 ± 1.19	0.53 ± 0.45	4.78 ± 8.09	3.75 ± 4.89	2.30 ± 2.78	4.62 ± 3.84	NS	NS	NS
Orchid mycorrhizal-Plant pathogen-Wood saprotroph	1.39 ± 1.73	1.37 ± 3.00	3.08 ± 5.15	3.31 ± 4.92	0.13 ± 0.06	0.18 ± 0.07	NS	NS	NS
Dung saprotroph	1.24 ± 0.69	3.97 ± 2.19	1.09 ± 0.87	2.43 ± 2.32	0.18 ± 0.02	0.38 ± 0.50	*	NS	NS
Endophyte-Undefined saprotroph	2.07 ± 1.70	0.99 ± 0.72	2.18 ± 2.73	2.71 ± 2.13	0.90 ± 1.00	1.34 ± 0.59	NS	NS	NS

Animal pathogen-Undefined saprotroph	2.66 ± 2.42	1.38 ± 2.94	3.37 ± 2.46	0.93 ± 0.61	0.71 ± 0.37	1.33 ± 1.48	**	NS	NS
Plant pathogen-Wood saprotroph	0.07 ± 0.02	0.20 ± 0.43	0.08 ± 0.05	0.08 ± 0.02	6.28 ± 4.11	13.17 ± 9.19	NS	**	**
Endophyte-Fungal/Lichen parasites-Plant patho-Wood saprotroph	2.69 ± 2.70	0.82 ± 0.78	1.35 ± 1.85	2.05 ± 1.94	0.74 ± 1.01	1.16 ± 0.49	NS	NS	*
Arbuscular mycorrhizal fungi	0.90 ± 0.61	1.14 ± 1.16	1.09 ± 0.73	1.18 ± 1.03	2.46 ± 1.51	1.55 ± 1.09	NS	NS	**
Soil saprotroph	0.71 ± 1.73	1.34 ± 0.94	1.29 ± 1.70	1.46 ± 1.41	0.04 ± 0.01	0.91 ± 0.06	NS	NS	NS

Endo = endophyte, patho = pathotroph. Significant levels: $^{*}P<0.05$; $^{**}P<0.01$; $^{***}P<0.001$; NS=Not significant.

Supplementary Table S10. Network-level topological features of the core mycobiome network of cultivated and wild pearl millet

	Cultivated plants	Wild plants
Average degree	7.54	6.97
Network diameter	9	10
Average path length	3.82	3.95
Graph density	0.03	0.04
Modularity	0.63	0.70
Clustering coefficient	0.50	0.55

Supplementary Table S11: Crop precedents and inputs used in the last year before the experiment for each experimental plot

Sites	Plots	Previous culture	Fertilization	Cooccurring plants
**Darou Mousty**	**P1**	Cowpea	Unfertilized	Striga<br>Grasses
	**P2**	Fallow land over 10 years old	Fertilized (NPK $_{15\text{-}10\text{-}10}$)	Weed-free field
**Dya**	**P3**	Groundnut	Unfertilized	Trees<br>Grasses<br>*Sesbania rostrata*
	**P4**	Groundnut	Fertilized (NPK $_{15\text{-}10\text{-}10}$)	-
	**P5**	Orchard	Unfertilized	Mango trees<br>Peppers<br>Cassava<br>Weed-free field<br>Millet plants already uprooted
**Nioro**	**P6**	Groundnut	Fertilized (NPK $_{15\text{-}10\text{-}10}$ and Urea)	Grasses

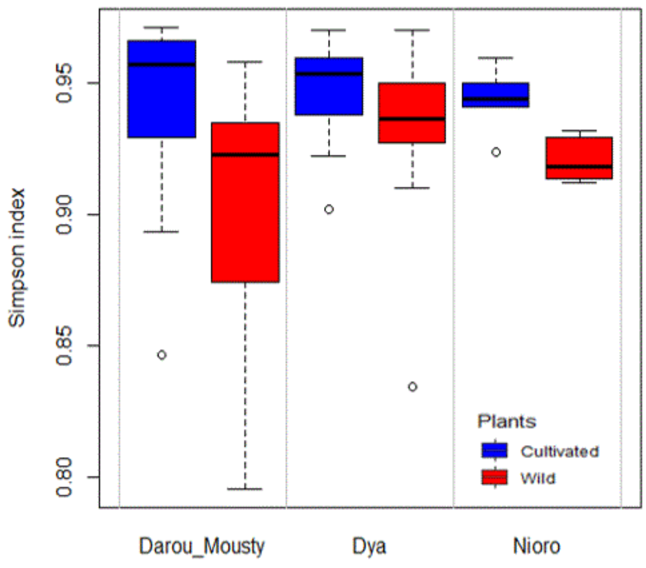

Supplementary Figure S1. Simpson diversity index of the fungal communities associated with cultivated and wild Pearl millet in the three studied sites (Darou-Mousty, Dya and Nioro). In the linear mixed effects (LME) model used to test the effect of plant type and site, plot was included as a random factor (see **Table 1**).

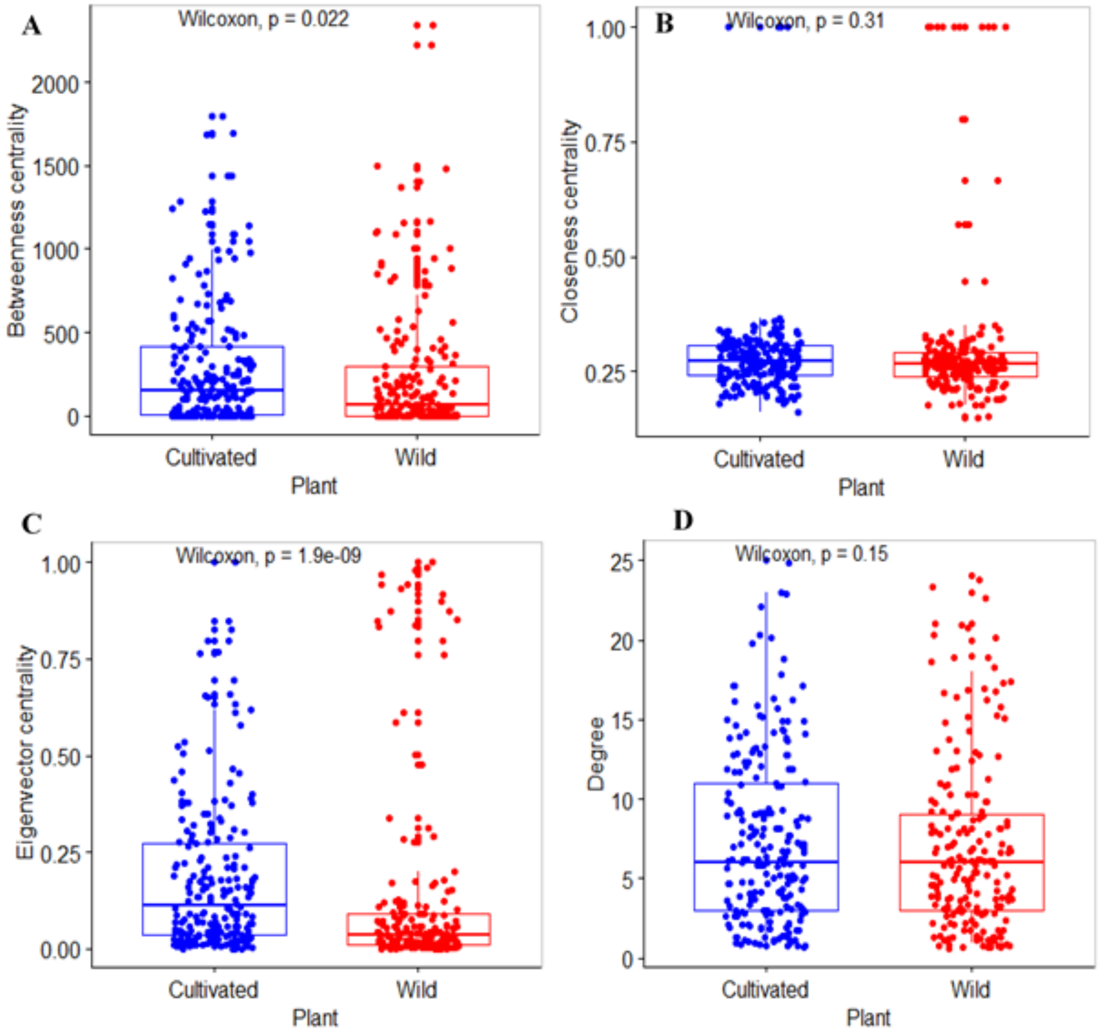

Supplementary Figure S2. Node-level topological features of the core mycobiome network of cultivated and wild pearl millet: (a) betweenness centrality, (b) closeness centrality, (c) eigenvector centrality, and (d) degree.

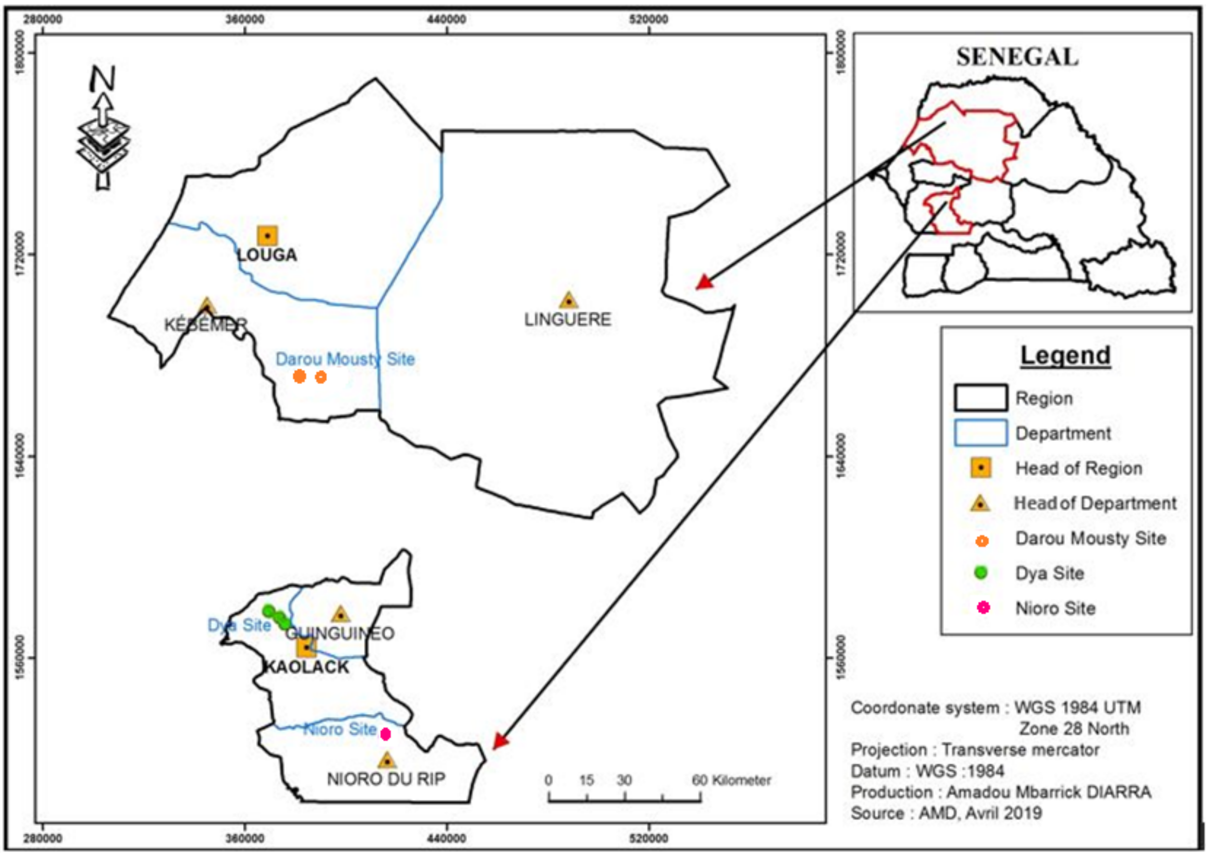

Supplementary Figure S3. Locations of the experimental sites

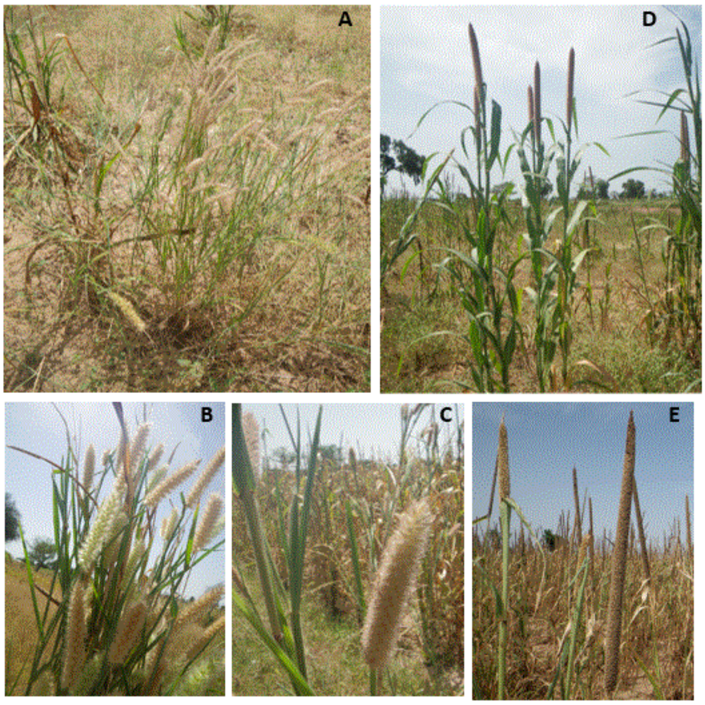

Supplementary Figure S4. Differences between cultivated and wild pearl millet. A) Bushy clumps of wild millet; B) Wild millet plant with its ears; C) Spikes of wild millet; D) Cultivated millet plant; E) Spikes of cultivated millet.
